# Uncovering the heterogeneity of the gut microbial taxa associated with the contents of different fatty acids in muscle with cecum luminal content and fecal samples from two pig populations

**DOI:** 10.3389/fmicb.2025.1575383

**Published:** 2025-04-30

**Authors:** Xiao Sun, Bin Yang, Congying Chen

**Affiliations:** National Key Laboratory of Pig Genetic Improvement and Germplasm Innovation, Jiangxi Agricultural University, Nanchang, China

**Keywords:** gut microbiome, fatty acids, two-part model association analysis, 16S rRNA gene sequencing, pigs

## Abstract

Fatty acids in pork are involved in cellular physiological functions and related to meat nutrition, tenderness, and flavor. Increasing evidences have suggested that short-chain fatty acids produced by the gut microbiota may affect host metabolism and energy utilization. However, the association between gut microbiota and long-chain fatty acids (LCFAs) in pork has been largely unknown. In this study, the microbial compositions of 243 cecum content samples from Erhualian pigs and 235 fecal samples from Bamaxiang pigs were determined by high throughput 16S rRNA gene sequencing. The contents of 12 LCFAs in longissimus dorsi (LD) muscle were also determined for all experimental pigs of both pig populations. We systematically evaluated the contribution of gut microbiota to the variations of muscle fatty acid contents from the *α*-diversity of gut microbiota, co-abundance groups (CAGs) of Amplicon Sequence Variants (ASVs), and fatty acid-associated bacterial taxa. We identified hundred ASVs and > 40 bacterial taxa that were significantly associated with muscle fatty acid contents in two pig populations. Different numbers and bacterial taxa associated with the content of specific LCFAs in muscle were detected between cecum luminal content and fecal samples, suggesting the heterogeneity of the specific LCFA-associated bacterial taxa between two gut locations. We uncovered some interesting associations between bacterial taxa and muscle fatty acid contents. The strongest association was observed between the ASV annotated to *Akkermansia* and the n-6/n-3 polyunsaturated fatty acid ratio (*p* = 6.45E-04, *Z* = −9.65). The gut microbiota could explain 1.47–4.62% variation of muscle contents of twelve fatty acids. The functional prediction analysis identified that the KEGG pathways related to the metabolisms of carbohydrate and lipids, and to fat digestion and absorption were positively associated with the contents of muscle fatty acids. However, adipocytokine signaling pathway and thermogenesis were negatively associated with muscle fatty acid contents. The results from this study provided the basic knowledge for improving the muscle fatty acid contents by regulating the gut microbiome.

## 1 Introduction

Pork is one of the most important red meats, which accounts for over a third of meats consumed by humans around the world. Fatty acids are important components of pork, which provide basic energy sources and are involved in cellular physiological functions and metabolisms in animals. Fatty acids are also related to meat nutrition, tenderness, and flavor (Wood et al., 2008). More and more attentions have been paid to the relationship of meat fatty acid compositions and contents with human health. For example, high ratios of omega-3 to omega-6 fatty acids (n-3/n-6) and polyunsaturated to monounsaturated fatty acids (PUFA/MUFA) are more favorable to human health (Jimenez-Colmenero et al., 2010). In general, meats contain a small amount of n-3 PUFAs and a relatively large amount of n-6 PUFAs (Simopoulos, 1998). These two PUFAs play a crucial role in cardiovascular diseases (Cao et al., 2024), such as dyslipidemia, diabetes, and hypertension. Low contents of n-3 PUFAs could strongly reduce the risk of cardiovascular diseases (Yang L. G. et al., 2016), such as cardiac arrhythmias. Previous studies also showed that the n-6/n-3PUFA ratio was associated with the reduced risk of diseases (Stene et al., 2003) and resulted in an enhance insulin secretion (Prada et al., 2023).

The composition and content of fatty acids in muscle are affected by multiple factors, including genetics (Cui et al., 2024) and various kinds of environmental factors. Previous studies revealed that the biosynthesis of n-3 and n-6 PUFAs is a complex process involved in many key enzymes and related genes (Yang et al., 2013). Increasing evidences have been suggested that the gut microbiota, as an important environmental factor, can influence host metabolism by modifying dietary nutrients. For instance, gut microbiota can increase blood glucose levels which are strongly linked to obesity (Ciubotaru et al., 2015). Likewise, gut microbiota can produce short-chain fatty acids (SCFAs) which can be used for the biosynthesis of glucose and lipids (Wolever et al., 1989; Topping and Clifton, 2001). Moreover, gut microbiota can activate intestinal gluconeogenesis (IGN) which regulates the synthesis of SCFAs and soluble fiber via gut-brain neural circuits (De Vadder et al., 2014). Substantial studies have revealed that the dysbacteriosis of gut microbiota affects host energy regulation and causes many metabolic syndromes, such as obesity (Ley et al., 2006), cardiovascular disease (O’Donnell et al., 2023), and type II diabetes (Baars et al., 2024). The association of gut microbiota with meat fatty acids were also found in pigs. Duroc × Landrace × Yorkshire pigs which were transplanted fecal microbiota from obese Ningxiang pigs had increased backfat thickness and intramuscular fat, as well as increased contents of saturated fatty acids in muscle (Yin et al., 2023). Gut microbiota can also mediate the production of some fatty acids. For example, *Fusimonas intestini* which colonized in the gut of humans and mice with obesity and hyperglycemia can produce long-chain fatty acids to promote diet-induced obesity (Takeuchi et al., 2023). However, how much is the contribution of gut microbiota to the composition and content of muscle fatty acids and which taxa in the gut microbiota were related to the contents of fatty acids, especially long-chain fatty acids (LCFA) in pork have been largely unknown.

Our objective in this study was to systematically evaluate the association between gut microbiota and muscle LCFA contents in two pig populations and identify microbial taxa influencing the content of various fatty acids in pork. Therefore, we first used the 16S ribosomal RNA (16S rRNA) gene sequencing to quantify the compositions of gut microbiota, and then revealed the contribution of gut microbiota to the content of fatty acids in longissimus dorsi (LD) muscle by applying the two-part model association analysis. The results should give important insights into the role of gut microbiota in muscular fatty acid contents in swine and provided basic knowledge for establishing the possible method regulating the gut microbiota to improve the contents of muscle fatty acids and to produce pork benefiting for human health.

## 2 Materials and methods

### 2.1 Experimental animals, phenotyping the contents of fatty acids in LD muscle, and microbial sample collection

A total of 243 Erhualian and 235 Bamaxiang pigs were used in this study. The two pig populations were raised in the same indoor conditions and were provided with the same commercial formula diet containing 14.20% crude protein, 3,150 Kcal/kg of digestible energy, and 0.78% lysine in the experimental pig farm of the National Key Laboratory of Pig Genetic Improvement and Germplasm Innovation, Nanchang, China. Ingredient compositions and nutrient levels of this commercial formula diet were described in detailed in our previous report (Zhou et al., 2025). Water was available *ad libitum* from nipple drinkers. The detailed information about feeding and management of experimental pigs were described in our previous study (Chen et al., 2018; He et al., 2019). All animals were slaughtered at the age of 300 ± 3 days (the body weight: 61.35 ± 9.10 kg for Bamaxiang, and 83.95 ± 13.70 kg for Erhualian) by bleeding after electrical stunning. Sex and slaughter batch were recorded detailedly.

Twelve types of fatty acids with 14 to 20 carbons were measured in LD muscle samples from two pig populations (Supplementary Table 1). The content of each fatty acid in tested samples was quantified using the methods described in our previous study (Guo et al., 2009; Yang et al., 2010; Yang et al., 2011; Zhang et al., 2016). The percentage of each fatty acid relative to total fatty acids was treated as phenotypic value and used for further analyses. The same measurement procedures were used for both populations. We also calculated the n-6/n-3 ratio in tested individuals and used for further analysis.

Fecal Samples were collected from the anus of Bamaxiang pigs within 12 h before the slaughter. For Erhualian pigs, luminal content samples were harvested from the cecum within 30 min after slaughter. All samples were deep frozen in liquid nitrogen. After transported to laboratory, all samples were stored at −80°C until utilization.

### 2.2 Microbial DNA extraction, 16S rRNA gene sequencing, and data processing

Microbial DNA was extracted from collected samples using QIAamp Fast DNA Stool Mini Kit following the manufacturer’s instructions (Qiagen, Germany) and stored at −20°C until utilization. DNA concentration for each sample was measured with a ND-1000 spectrophotometer (Nanodrop Technologies, USA). The quality and integrity of DNA samples were determined by electrophoresis with 0.8% agarose gel. The V4 hypervariable region of 16S rRNA gene was selected and amplified by the fusion primers 515F (5’-GTGCCAGCMGCCGCGGTAA-3′) and 806R (5’-GGACTACHVGGGTWTCTAAT-3′) under the melting temperature of 56°C with 30 cycles. The sequencing of PCR amplicons was performed with a 250-bp paired-end strategy on an Illumina MiSeq platform (Illumina, USA) according to the manufacturer’s protocol. Raw sequencing data were processed with the QIIME2 software (Quantitative Insights Into Microbial Ecology version 2, v2024.10.1) (Hall and Beiko, 2018). Clean sequence reads were further assigned to Amplicon Sequence Variants (ASVs) using DADA2 (Callahan et al., 2016). According to the filtration standards report by Fu et al. (2015), ASVs occupying ≥ 0.005% of total sequence reads and existed in at least 1.0% of tested samples were used for further statistical analysis. The taxonomic annotations of ASVs were performed using the QIIME2 based on the SILVA database (v.138). The *α*-diversity of gut microbiota was assessed using the QIIME2. The relative abundances of microbial taxa at different taxonomic levels were calculated by summarizing the abundances of ASVs distributing in each taxonomy.

### 2.3 Association analysis between microbial taxa and the contents of LCFAs in muscle by a two-part model

Considering the effect of sex and slaughter batch on muscle LCFA contents, the phenotypical values of muscle LCFA contents were adjusted for the effect of sex and slaughter batch. The residuals were used for further association analysis with the relative abundances of bacteria. Based on the characterization of gut microbial composition and abundance, a two-part model was used to identify microbial taxa associated with the contents of muscle fatty acids (Fu et al., 2015). In brief, the binary model interpreted a binomial analysis that tested for association of detecting a microbe with phenotypic values of fatty acids. Binary data 0 or 1 was used for undetected or detected microbe in each sample in the study. The binary model was described as: y = β1b + e, where y represents the trait value (fatty acid content) per individual after adjusting for the gender and slaughter batch, β1 is the estimated effect of undetected or detected microbe, b is a binary feature and e refers to the residuals. Quantitative analysis tested for the association between phenotypic values of fatty acids and the abundance of present microbial taxa. The abundances (q) of the microbiota were log_10_ transformed for read count per individual before the next step. The quantitative model was described as: y = β2q + e, where β2 is the estimated effect for the abundance, q represents the abundance of the microbiota and e is the residuals.

*p* value of the meta-analysis was derived using an unweighted *Z* method to further combine the effect of binary and quantitative analysis. The final associated *p* value per microbe-phenotype pair was from the minimum of *p* values from the binary model, quantitative analysis, and meta-analysis. The *Z* score was obtained based on the *Z* distribution. Positive and negative values meant positive and negative associations, respectively. To eliminate the skew on the minimization of *p* values in three parts of association analysis, 1,000 × permutation tests were performed to control the false discovery rate (FDR). We set the FDR ≤ 0.05 as the significance threshold. *p* value from the binary model, quantitative analysis, and meta-analysis accounted for the significant effect of the presence/absence of microbe, abundance level of microbe, and both presence and abundance of microbe on the phenotypic values of fatty acids, respectively. This analysis was performed on both ASVs and bacterial taxa using the R software (v 4.2.1).

### 2.4 Co-abundance network analysis

The correlations among ASVs were calculated and converted into the distance matrix based on the abundance of ASVs by FastSpar (v1.0.0) (Watts et al., 2019). And then, ASVs in the matrix were clustered into co-abundance groups (CAGs) by the hclust function (ward. D2 algorithm) in R package (Murtagh and Legendre, 2014). The determination of CAGs was based on the permutational multivariate ANOVA (PerMANOVA) analysis with 999 permutations (*p* < 0.05). The Spearman correlations between CAGs and the contents of muscle fatty acids were calculated using the average abundance of all ASVs in each CAG. The co-abundance network was visualized with Cytoscape (v3.10.0) (Shannon et al., 2003).

### 2.5 Functional prediction of gut microbiome based on 16S rRNA gene sequencing data

PICRUSt2 (v2.5.3) was applied to predict potential functional capacity of gut microbiome based on 16S rRNA gene sequencing data (Douglas et al., 2020). The ASV abundance table was imported into the PICRUSt2. Functional items and their abundances in each sample were predicted and calculated according to the Kyoto Encyclopedia of Genes and Genomes (KEGG) catalog. The annotated genes were assigned into KEGG pathways for further analysis (Kanehisa et al., 2025). The correlations between KEGG pathways and the content of each fatty acid in muscle were analyzed by the two-part model as described above.

### 2.6 Statistical analysis

The Spearman correlation analyses among LCFAs and between the *α*-diversity indices and muscle LCFA contents were performed by the *corr.test* in R package (v 4.2.1). The correlation coefficient (R) and linear regression line between CAGs and LCFA contents were calculated using the *stat_cor* and *stat_smooth* functions. The correlation results were visualized using R (*corrplot* and *ggplot2* packages). The association of the *β*-diversity of cecum and fecal microbiota (based on the Bray-Curtis distance matrix) with muscle LCFA contents were determined by PERMANOVA analysis with 9,999 permutations using adonis2 in vegan. *p* < 0.05 was set as the significance level.

To estimate the contribution of gut microbiome to the phenotypic variation of fatty acid contents, we performed a 100 times cross-validation. The data was randomly split into a 70% discovery set and a 30% validation set. And then, an additive model was used to estimate the contribution of the gut microbiome to the variance of fatty acid contents (*r*_m_) in the validation set:


$$r_{m}=\sum_{j=1}^{n}\left(\beta_{1}+b_{j}+\beta_{2}q_{j\text{.}}\right)$$

Where n is the number of significantly associated ASVs at a certain *p* value. β_1_ and β_2_ are the estimated effect sizes of binary and quantitative features. The explained variance was represented by the average value of the squared correlation coefficient (R^2^) in a 100 times regression analysis.

## 3 Results

### 3.1 The contents of fatty acids in LD muscle of Erhualian and Bamaxiang pigs

**Figure 1:**
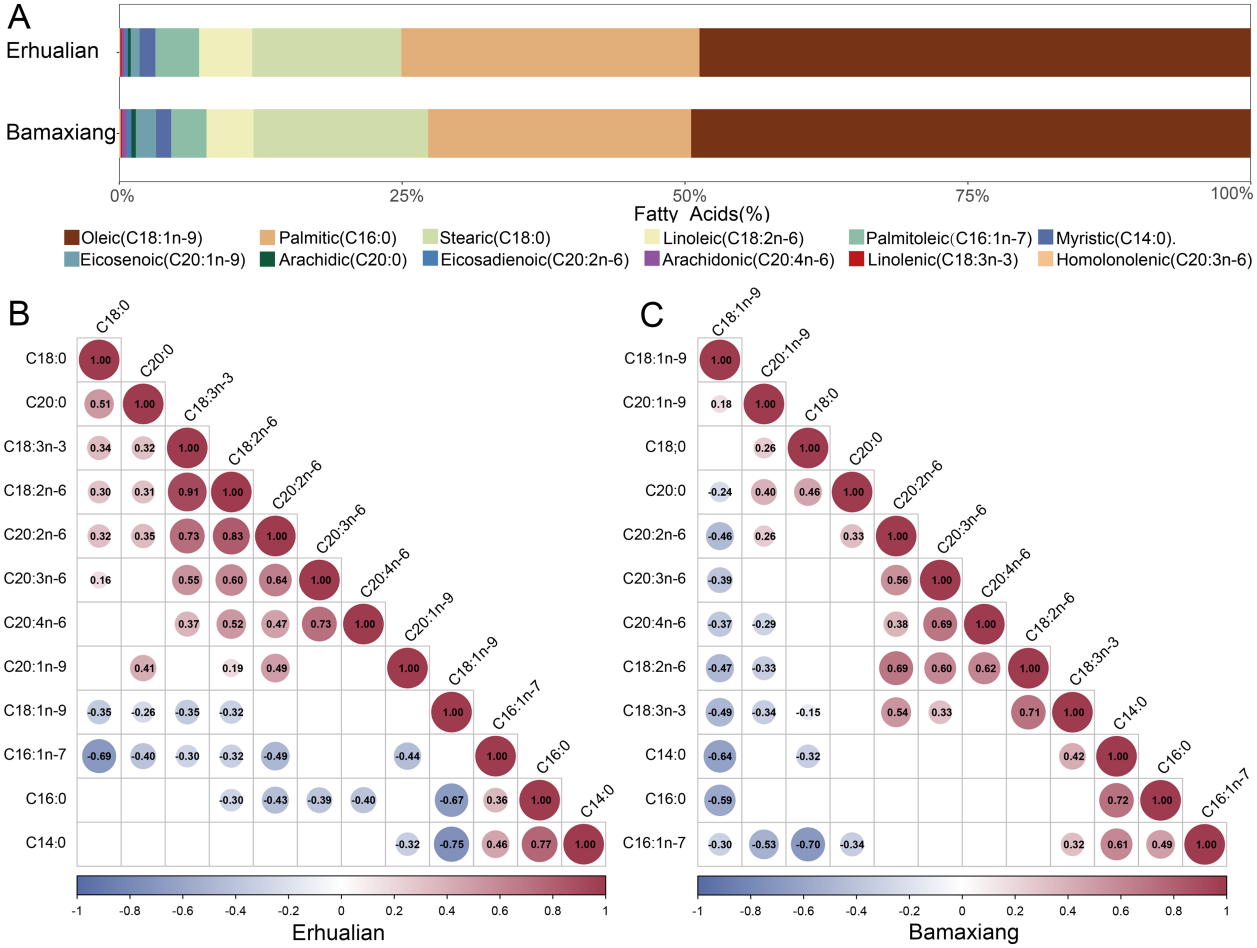
Contents and correlations of 12 fatty acids in longissimus dorsi muscle of Erhualian and Bamaxiang pigs. **(A)** Summary statistic for fatty acid composition in longissimus dorsi muscle of Erhualian and Bamaxiang pigs. Different colored boxes represented different types of 12 fatty acids. **(B,C)** The correlations among 12 fatty acids in longissimus dorsi muscle of Erhualian **(B)** and Bamaxiang pigs **(C)**. The numbers indicate spearman correlation coefficients between pair-wise fatty acids. The size of circle represents the strength of correlations. Red cycles indicate positive correlations and blue cycles show negative correlations.

The most abundant fatty acid was oleic acid (C18:1n-9), followed by palmitic acid (C16:0) and stearic acid (C18:0) in both pig populations (Figure 1A). These three fatty acids accounted for 90% of the total content of fatty acids in muscle samples. Nevertheless, fatty acids with 20 carbons were less abundant in tested samples. For more details, in LD muscle samples of Erhualian pigs, there were 24 pairs of fatty acids showing significant positive correlation (*p* < 0.05), among which 23 pairs showed extremely positive correlations (*p* < 0.01). The strongest positive correlation was observed between linoleic acid (C18:2n-6) and linolenic acid (C18:3n-3) (*r* = 0.91), and followed by the correlations between linoleic acid (C18:2n-6) and eicosadienoic acid (C20:2n-6) (*r* = 0.83), and between myristic acid (C14:0) and palmitic acid (C16:0) (*r* = 0.77). Simultaneously, 17 pairs of fatty acids showed extremely negative correlations (*p* < 0.01). The most significant negative correlation was identified between oleic acid (C18:1n-9) and myristic acid (C14:0) (*r* = −0.75), followed by the negative correlations between palmitoleic acid (C16:1n-7) and stearic acid (C18:0) (*r* = −0.69), and between oleic acid (C18:1n-9) and palmitic acid (C16:0) (*r* = −0.67) (Figure 1B).

The similar conditions were also found in Bamaxiang pigs. We observed 20 pairs of positive correlations and 16 pairs of negative correlations among fatty acids in LD muscle samples of Bamaxiang pigs (*p* < 0.05), including 33 pairs of correlations achieving extreme significance level (*p* < 0.001). Similar to that in Erhualian pigs, the strongest positive correlation was identified between myristic acid (C14:0) and palmitic acid (C16:0) (*r* = 0.72), followed by the correlations between linoleic acid (C18:2n-6) and linolenic acid (C18:3n-3) (*r* = 0.71), and between homolonolenic acid (C20:3n-6) and arachidonic acid (C20:4n-6) (*r* = 0.69), while the strongest negative correlation was found between palmitoleic acid (C16:1n-7) and stearic acid (C18:0) (Figure 1C).

### 3.2 The compositions of gut microbiota in Erhualian and Bamaxiang pigs

The bacterial compositions in luminal content samples of 243 Erhualian pigs and fecal samples of 235 Bamaxiang pigs were described in detail in our previous publications (Fang et al., 2017; He et al., 2019). In brief, in luminal content samples of Erhualian pigs, a total of 4,177 ASVs were obtained. After quality control (see methods), 983 ASVs were left for further analysis. There were 22 ASVs (2.2%) were presented in > 90% of samples, while 846 ASVs (86.1%) were found in < 50% of samples. At the taxonomic level, a total of 141 taxa were obtained. Among them, 23 (16.3%) taxa were found in > 90% of samples and 50.4% of taxa were identified in < 50% of samples. The abundances of *unclassified Lachnospiraceae*, *Alloprevotella*, *unclassified Prevotellaceae*, *Treponema*, and *Prevotellaceae_NK3B31_group* were listed in the top five. These five taxa accounted for 34.75% of total abundance. We obtained 2,481 ASVs in 235 fecal samples of Bamaxiang pigs. A total of 873 ASVs passed the quality control. There were 39 ASVs (4.5%) that were presented in > 90% of samples and 81.2% of ASVs that existed in <50% of samples. After the taxonomy annotation, 137 taxa were obtained and 48 (35.0%) taxa were found in > 90% of samples and 49 (35.8%) taxa were identified in < 50% of samples. The bacterial taxa whose abundances were listed in the top five included *Treponema* (15.94% in relative abundance), *UCG-005* (6.92%), *Rikenellaceae_RC9_gut_group* (6.18%), *unclassified Lachnospiraceae* (5.13%), and *unclassified Prevotellaceae* (4.73%).

### 3.3 Association of the diversity and co-abundance groups (CAGs) of gut microbiota with the contents of fatty acids in LD muscle

**Figure 2:**
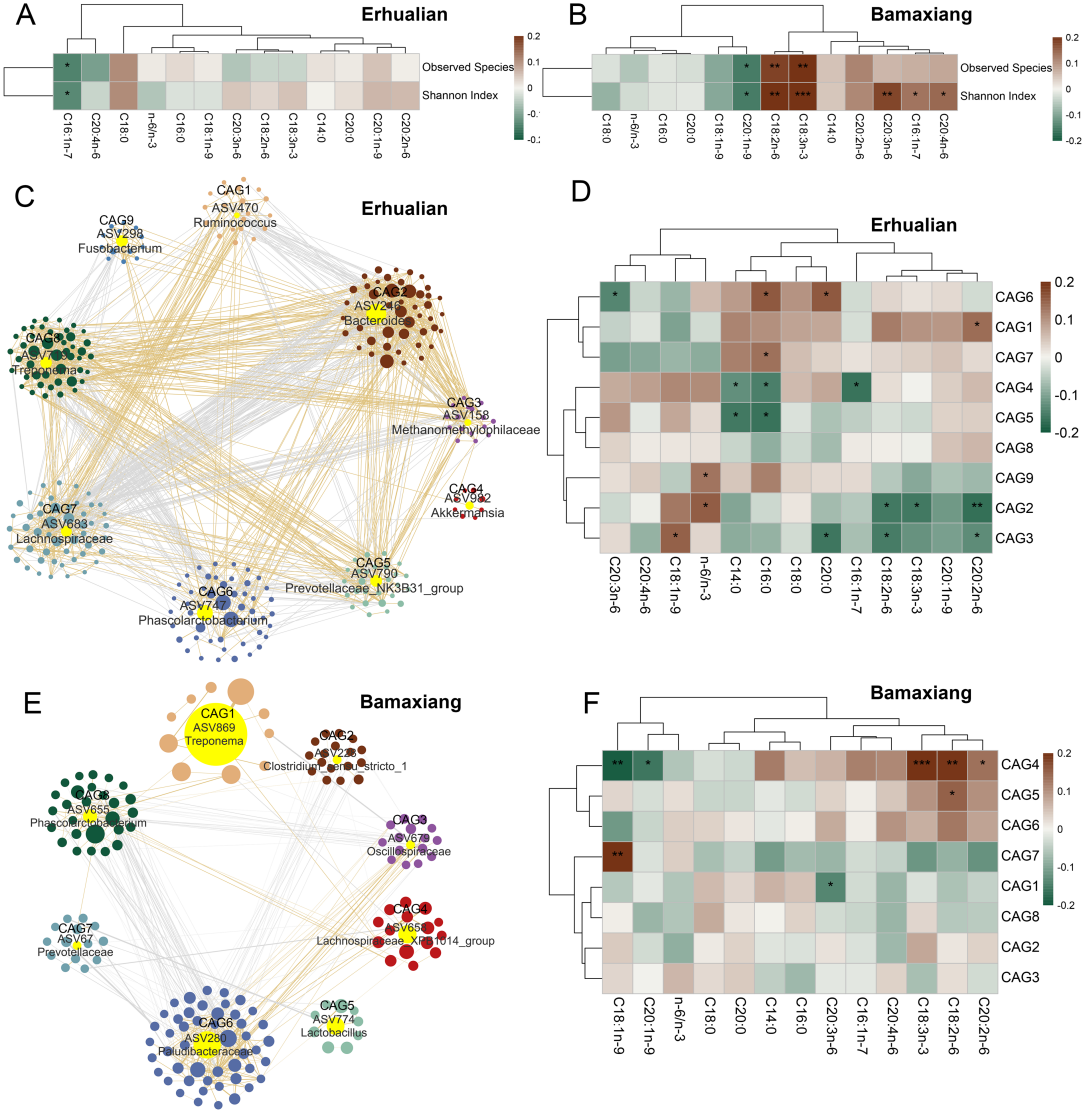
Correlations of the *α*-diversity and co-abundance groups of amplicon sequence variants (ASVs) of gut microbiota with the contents of fatty acids in longissimus dorsi muscle of Erhualian and Bamaxiang pigs. **(A,B)** The correlations of observed species and Shannon index with the contents of 12 fatty acids and n-6/n-3 ratio in Erhualian **(A)** and Bamaxiang pigs **(B)**. **(C)** Co-abundance network of ASVs constructed in cecum luminal content samples of Erhualian pigs. **(D)** The correlations of CAGs with the contents of 12 fatty acids and n-6/n-3 ratio in Erhualian pigs. **(E)** Co-abundance network of ASVs constructed in feces samples of Bamaxiang pigs. **(F)** The correlations of CAGs with the contents of 12 fatty acids and n-6/n-3 ratio in Bamaxiang pigs.

We first analyzed the associations between the *α*-diversity of gut microbiota and muscle fatty acid contents. We only found that palmitoleic acid (C16:1n-7) was negatively correlated with the α-diversity indices of observed species and Shannon index in the Erhualian pigs (Figure 2A). In Bamaxiang pigs, we observed the significantly positive correlations of linoleic acid (C18:2n-6) and linolenic acid (C18:3n-3) with the observed species (*p* < 0.01), whereas eicosenoic acid (C20:1n-9) showed a negative correlation with observed species (*p* < 0.05). We also identified that palmitoleic acid (C16:1n-7), linoleic acid (C18:2n-6), linolenic acid (C18:3n-3), homolonolenic acid (C20:3n-6), and arachidonic acid (C20:4n-6) were positively correlated with the Shannon index (*p* < 0.05), while eicosenoic acid (C20:1n-9) was negatively correlated with the Shannon index (Figure 2B). Regarding the correlation between *β*-diversity of microbial composition and muscle LCFA content, LCFA levels explained a substantial 4.29% of the variance in β-diversity within Erhualian pigs. Specifically, C20:3n-6 and the n-6/n-3 ratio contributed 1.23 and 0.94% to this variance, respectively (*p* = 0.006 and *p* = 0.016; Supplementary Figure 1). In contrast, although LCFAs collectively accounted for 3.19% of the β-diversity variance in Bamaxiang pigs’ fecal microbiota, no individual LCFA exhibited statistically significant effects.

We then evaluated the associations of CAGs with muscle fatty acid contents. In Erhualian pigs, 983 ASVs that passed quality control were clustered into nine CAGs (Figure 2C). CAG1 and CAG7 were positively correlated with eicosadienoic acid (C20:2n-6) and palmitic acid (C16:0), respectively (*p* < 0.05). CAG2 were negatively correlated with linoleic acid (C18:2n-6), linolenic acid (C18:3n-3), and eicosadienoic acid (C20:2n-6) (*p* < 0.05). CAG4 and CAG5 were negatively correlated with both myristic acid (C14:0) and palmitic acid (C16:0). In addition, CAG4 was also negatively correlated with palmitoleic acid (C16:1n-7) (*p* < 0.05). And CAG3 was negatively correlated with linoleic acid (C18:2n-6), arachidic acid (C20:0), and eicosadienoic acid (C20:2n-6) (*p* < 0.05), while it showed a positive correlation with oleic acid (C18:1n-9) (*p* < 0.05). CAG6 was positively correlated with palmitic acid (C16:0) and arachidic acid (C20:0) (*p* < 0.05), but negatively correlated with homolonolenic acid (C20:3n-6) (*p* < 0.05). Among these nine CAGs, only CAG2 and CAG9 in Erhualian pigs showed a positive correlation with the n-6/n-3 ratio (Figure 2D). In Bamaxiang pigs, 873 ASVs were clustered into eight CAGs by co-abundance network analysis, significant correlations between CAGs and muscle fatty acid contents were significantly less than that identified in Erhualian pigs (Figures 2D–F). CAG4 was positively correlated with linoleic acid (C18:2n-6), linolenic acid (C18:3n-3), and eicosadienoic acid (C20:2n-6) (*p* < 0.05), but negatively correlated with oleic acid (C18:1n-9) (*p* < 0.01) and eicosenoic acid (C20:1n-9) (*p* < 0.05). In contrast, CAG5 and CAG7 were positively correlated with linoleic acid (C18:2n-6) and oleic acid (C18:1n-9), respectively (*p* < 0.05), while CAG1 was negatively correlated with homolonolenic acid (C20:3n-6) (*p* < 0.05) (Figure 2F).

### 3.4 Association of bacterial taxa with the contents of fatty acids in LD muscle

**Figure 3:**
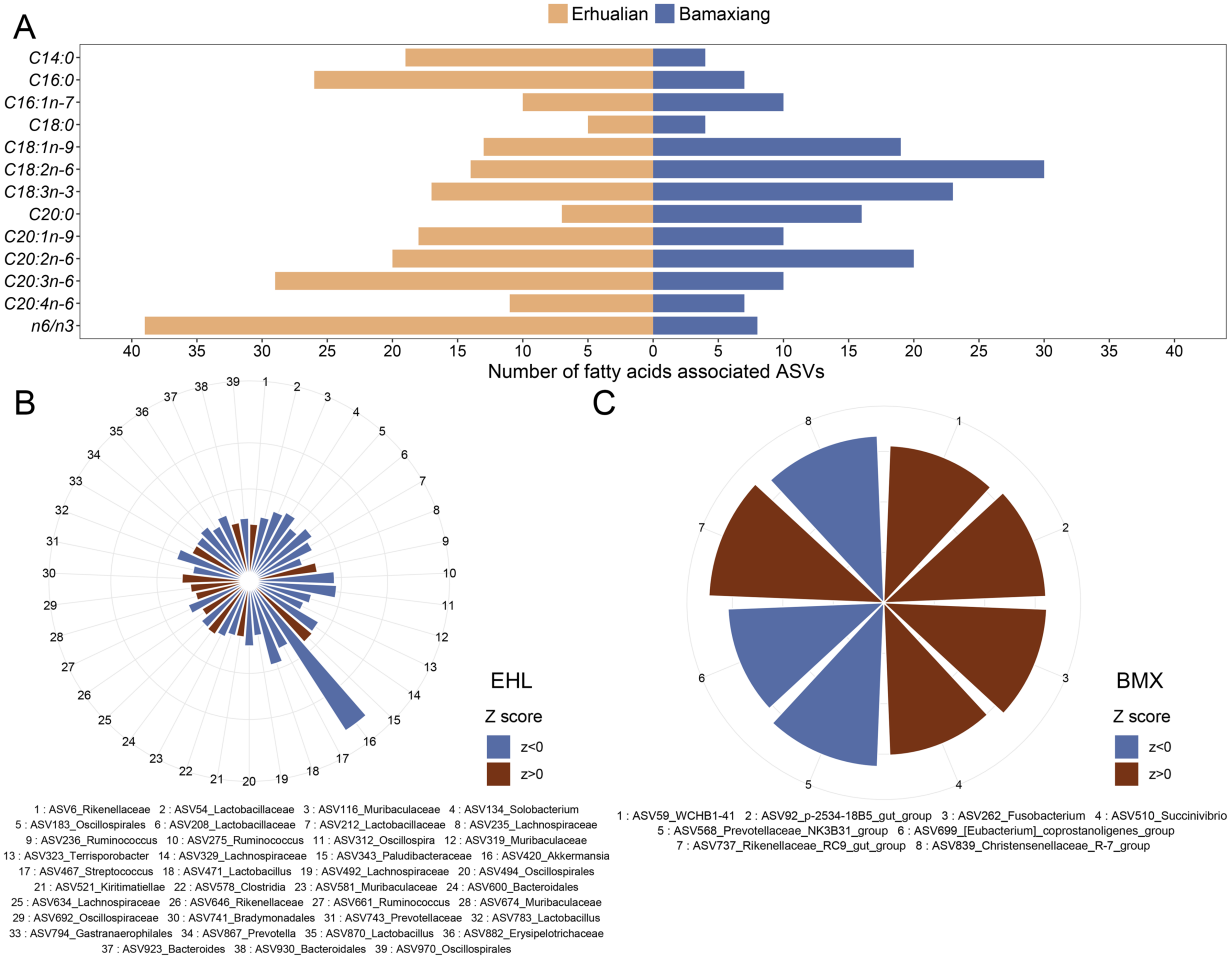
Associations of amplicon sequence variants (ASVs) of gut microbiota with the contents of fatty acids in longissimus dorsi muscle of Erhualian and Bamaxiang pigs. **(A)** Summary statistic for the numbers of ASVs associated with each fatty acid identified in two pig populations at the FDR < 0.05 level. **(B,C)** Significant associations of ASVs with the n-6/n-3 polyunsaturated fatty acid ratio in Erhualian **(B)** and Bamaxiang pigs **(C)**. Brown bars indicate positive associations (*Z* score > 0) and blue bars represent negative correlations (*Z* score < 0). Circles indicate the scale of absolute *Z* score from 1 to 10. The statistical analysis was performed by a two-part model. False discover rate (FDR) < 0.05 was set as the significance threshold.

**Figure 4:**
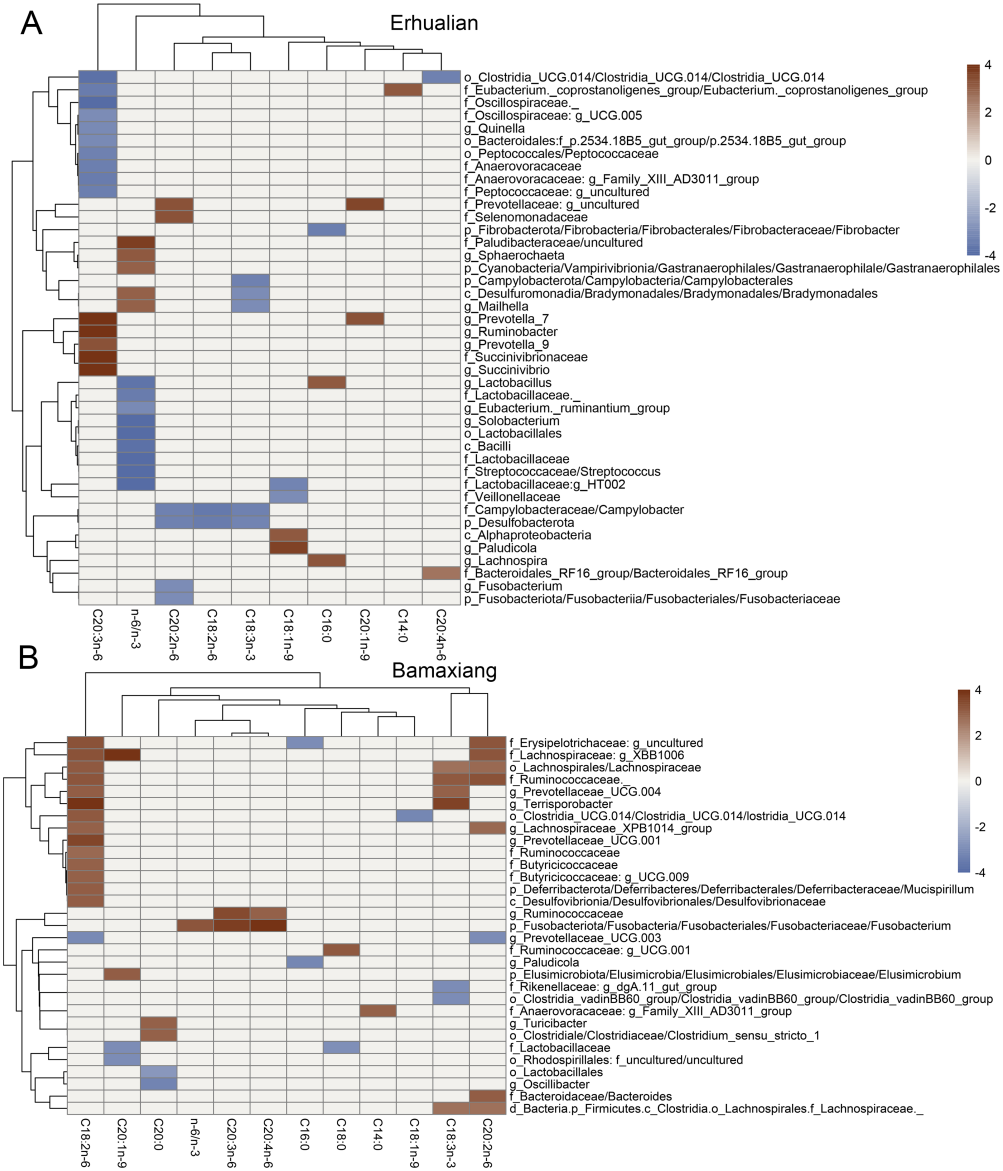
Heatmaps showing the associations of bacterial taxa with the contents of fatty acids in longissimus dorsi muscle of Erhualian and Bamaxiang pigs. **(A)** Erhualian pigs. **(B)** Bamaxiang pigs. Each sector is indicated by a color gradient from blue (negative associations) to red (positive associations). The grey sector indicate that the associations did not achieve significance level. The statistical were performed by a two-part model. False discover rate (FDR) < 0.05 was set as the significance threshold.

The associations between the contents of fatty acids in LD muscle and ASVs were analyzed by the two-part model. At the significance level of FDR < 0.05, a total of 189 ASVs were found to show significant associations with muscle fatty acid contents in Erhualian pigs, including 29 ASVs that were significantly associated with homolonolenic acid (C20:3n-6), 26 ASVs with palmitic acid (C16:0), 20 ASVs with eicosadienoic acid (C20:2n-6), 19 ASVs with myristic acid (C14:0), and 18 ASVs with eicosenoic acid (C20:1n-9) (Figure 3A; Supplementary Table 1). Among them, ASV811 annotated to Lachnospiraceae were simultaneously associated with linoleic acid (C18:2n-6), linolenic acid (C18:3n-3), eicosadienoic acid (C20:2n-6), and homolonolenic acid (C20:3n-6); ASV958 annotated to *Muribaculaceae* were simultaneously correlated with stearic acid (C18:0), linoleic acid (C18:2n-6), linolenic acid (C18:3n-3), and C20:2n-6. Specially, there were 39 ASVs that were significantly associated with n-6/n-3 ratio in Erhualian pigs (FDR < 0.05), including 11 ASVs that were detected by the binary model (presence/absence), six ASVs by quantitative model, and 22 ASVs by meta-analysis. It was worth noting that the ASV420 annotated to *Akkermansia* showed a negative association with the n-6/n-3 ratio and this was the strongest association identified in this study (*p* = 6.45E-04, *Z* score = −9.65) (Figure 3B). At the taxonomic level, we identified 40 significant associations that were involved in 32 unique bacterial taxa with muscle fatty acid contents at the significant threshold of FDR < 0.05. Among these associations, 15 taxa were significantly associated with homolonolenic acid (C20:3n-6), including Succinivibrionaceae, *Prevotella_7*, *Prevotella_9*, *Ruminobacter*, and *Succinivibrio* which showed positive associations. Six taxa were found to be significantly associated with eicosadienoic acid (C20:2n-6), among which uncultured Prevotellaceae and Selenomonadaceae were positively associated with eicosadienoic acid, while *Campylobacter*, *Fusobacterium*, Desulfobacterota, and Fusobacteriaceae were negatively associated with eicosadienoic acid. Five bacterial taxa were negatively associated with linolenic acid (C18:3n-3), including *Bradymonadales*, *Campylobacter*, *Mailhella*, Campylobacterales, and Desulfobacterota. We found that most of bacterial taxa negatively associated with linolenic acid belonged to Firmicutes, while taxa from Bacteroidales and Proteobacteria mainly showed positive associations with linolenic acid. A total of 14 bacterial taxa were identified to be significantly associated with the n-6/n-3 ratio in Erhualian pigs, including five taxa positively associated with the n-6/n-3 ratio and nine taxa negatively associated with the n-6/n-3 ratio. For example, Paludibacteraceae was positively associated with the n-6/n-3 ratio. This was consistent with that the ASV343 annotated to Paludibacteraceae were positively associated with the n-6/n-3 ratio. Most of the taxa negatively associated with the n-6/n-3 ratio belonged to Lactobacillales (Figure 4A; Supplementary Table 3). To determine the key bacterial taxa affecting the n-6/n-3 ratio, we combined the n-6/n-3 ratio-associated taxa that were detected by both two-part model and co-abundance network analyses. A total of eight n-6/n-3 ratio-associated ASVs were detected, including six ASVs belonging to Bacteroidales, such as Paludibacteraceae, Rikenellaceae, and Muribaculaceae, and two ASVs belonging to Lachnospiraceae and Oscillospiraceae (Supplementary Table 4).

In Bamaxiang pigs, we identified 121 ASVs that were associated with the contents of LCFAs in LD muscle at FDR < 0.05, including 30 ASVs significantly associated with linoleic acid (C18:2n-6), 23 ASVs with linolenic acid (C18:3n-3), 20 ASVs with eicosadienoic acid (C20:2n-6), 19 ASVs with oleic acid (C18:1n-9), and 16 ASVs with arachidic acid (C20:0) (Figure 3A; Supplementary Table 2). We found that the ASV352 annotated to *Prevotellaceae_UCG-001* and the ASV853 annotated to Lachnospiraceae were simultaneously associated with oleic acid (C18:1n-9), linoleic acid (C18:2n-6), and linolenic acid (C18:3n-3). We identified eight ASVs associated with the n-6/n-3 ratio in Bamaxiang pigs (Figure 3C). These ASVs were annotated to *Christensenellaceae_R-7_group*, *WCHB1-41* (Kiritimatiellae), *Rikenellaceae_RC9_gut_group*, *Fusobacterium*, *Prevotellaceae_NK3B31_group*, *p-2534-18B5_gut_group* (Bacteroidales), *Succinivibrio*, and *[Eubacterium]_coprostanoligenes_group*. At the taxonomic level, a total of 48 significant associations related to 31 unique bacterial taxa were identified for the contents of LCFAs in LD muscle. Among these associations, 15 taxa were significantly associated with linoleic acid (C18:2n-6), including *Prevotellaceae_UCG.003* that was negatively associated with linoleic acid and 14 taxa positively associated with linoleic acid. Most of these positively associated taxa belonged to Prevotellaceae, Lachnospiraceae, Butyricicoccaceae, and Ruminococcaceae. There were seven bacterial taxa that were significantly associated with linolenic acid (C18:3n-3). Among them, five taxa including *Prevotellaceae_UCG.004*, Lachnospiraceae, Ruminococcaceae, and *Terrisporobacter* were positively associated with linolenic acid. There were eight taxa significantly associated with eicosadienoic acid (C20:2n-6), including seven taxa that mostly belonged to Lachnospiraceae and were positively associated with eicosadienoic acid. Both Ruminococcaceae and *Fusobacterium* were positively associated with arachidonic acid (C20:4n-6) and homolonolenic acid (C20:3n-6). Different from the results in Erhualian pigs, we only identified that *Fusobacterium* was significantly associated with the n-6/n-3 ratio (positive) in Bamaxiang pigs (Figure 4B; Supplementary Table 3).

Finally, we compared LCFA-associated bacterial taxa between Erhualian and Bamaxiang pigs. At FDR ≤ 0.05, one ASV in each population (designated ASV66 in Erhualian and ASV551 in Bamaxiang) within the genus *Ruminococcus* exhibited positive correlations with linoleic acid (C18:2n-6), while a *Bacteroides*-affiliated ASV (ASV59 in Erhualian vs. ASV56 in Bamaxiang) showed positive associations with eicosadienoic acid (C20:2n-6). Notably, no taxa demonstrated consistent associations with identical LCFAs across both populations. At a relaxed threshold (FDR ≤ 0.1), the class *Clostridia vadinBB60_group* displayed negative correlations with linolenic acid (C18:3n-3) in both breeds. Intriguingly, *Campylobacter* exhibited divergent associations with linoleic acid (C18:2n-6) - negative in Erhualian versus positive in Bamaxiang pigs. Similarly, *Clostridia-UCG.014* showed opposing associations with arachidonic acid (C20:4n-6) between breeds (negative in Erhualian, positive in Bamaxiang). This bidirectional pattern likely stems from strain-level variations within *Campylobacter* and *Clostridia-UCG.014*, as 16S rRNA gene sequencing lacks sufficient resolution for precise taxonomic discrimination.

### 3.5 Predicted function capacity of gut microbiome related to the contents of fatty acids in LD muscle

**Figure 5:**
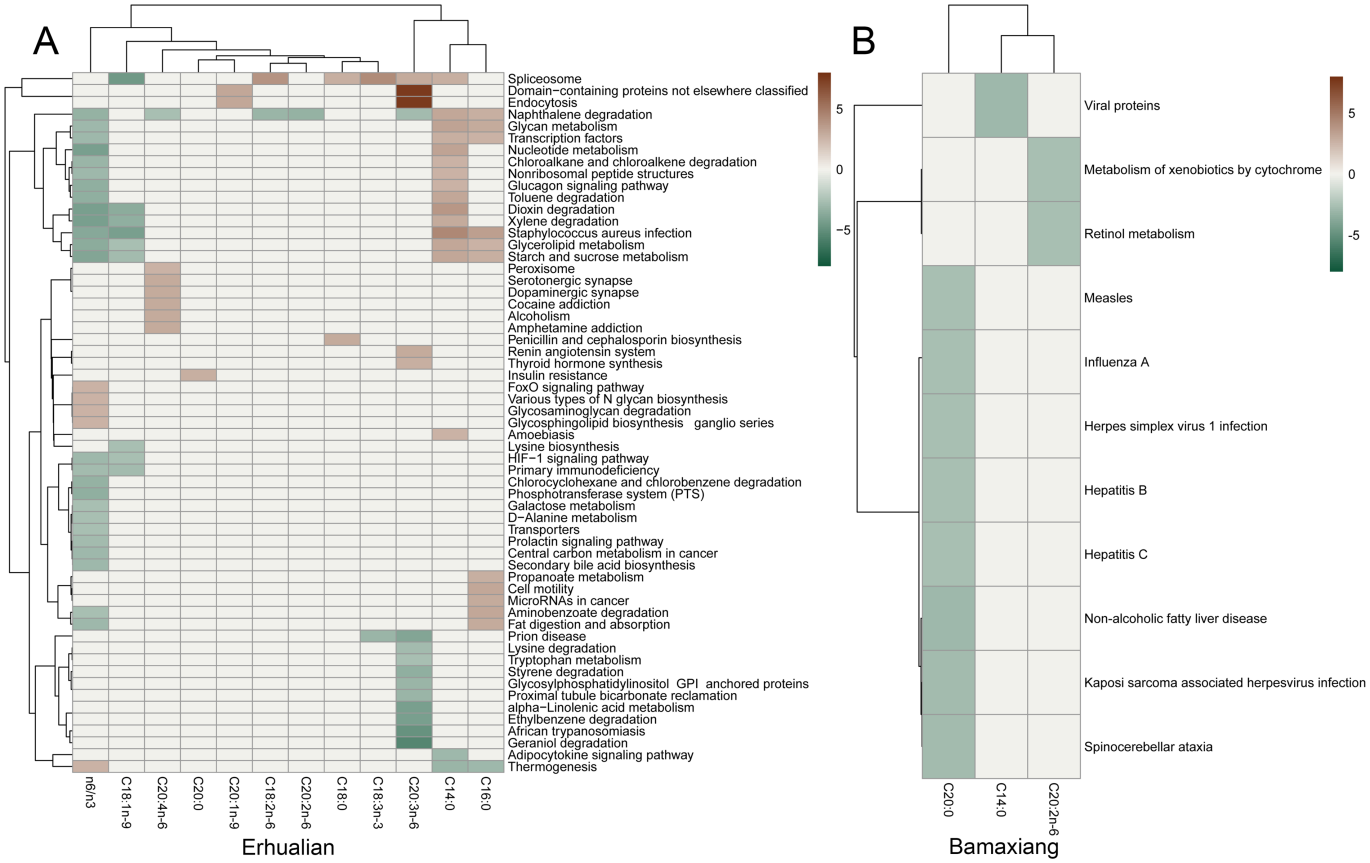
KEGG pathways predicted with 16S rRNA gene sequencing data and showing the associations with the contents of fatty acids and the n-6/n-3 polyunsaturated fatty acid ratio in Erhualian **(A)** and Bamaxiang **(B)** pigs. Each sector is indicated by a color gradient from green (negative associations) to dark red (positive associations). The grey sector indicate that the associations did not achieve significance level. The correlations between KEGG pathways and the content of each fatty acid in muscle were analyzed by the two-part model. False discover rate (FDR) < 0.05 was set as the significance threshold.

We predicted potential functional capacities of the gut microbiota and identified functional capacities associated with the contents of fatty acids in LD muscle. As shown in Figure 5A, in Erhualian pigs, at the significant threshold of FDR < 0.05, a total of 17 KEGG pathways were significantly associated with myristic acid (C14:0), including 15 pathways positively associated with myristic acid, such as the metabolisms of nucleotide, glycerolipid, glycan, and starch and sucrose, the degradations of naphthalene, xylene, dioxin, and chloroalkane and chloroalkene, and glucagon signaling pathway. Two pathways were negatively correlated with myristic acid, including adipocytokine signaling pathway and thermogenesis. The contents of palmitic acid (C16:0) in LD muscle samples were positively associated with starch and sucrose metabolism, propanoate metabolism, glycerolipid and glycan metabolism, aminobenzoate degradation, and fat digestion and absorption, but negatively correlated with thermogenesis. We also observed that homolonolenic acid (C20:3n-6) were negatively associated with *α*-linolenic acid metabolism, tryptophan metabolism, lysine degradation, and ethybenzene degradation. There were also 31 KEGG pathways that were significantly associated with n-6/n-3 ratio (Figure 5A), including five positive associations and 25 negative associations. Particularly, the pathways related to the metabolisms of glycan, galactose, glycerolipid, alanine, nucleotide, starch and sucrose, secondary bile acid biosynthesis fat digestion and absorption, and so on were negatively associated with the n-6/n-3 ratio. However, FoxO signaling pathway, various types of N-glycan biosynthesis, glycosaminoglycan degradation, and glycosphingolipid biosynthesis were positively correlated with the n-6/n-3 ratio. However, we only found 11 KEGG pathways that were significantly associated with fatty acid contents in fecal samples of Bamaxiang pigs. All these associations were negative, including nine pathways associated with arachidic acid (C20:0) (most of these pathways were related to diseases), two pathways related to eicosadienoic acid (C20:2n-6), and one pathway associated with myristic acid (C14:0). Strangely, we did not identify any KEGG pathway associated with n-6/n-3 ratio in Bamaxiang pigs (Figure 5B).

### 3.6 Contribution of the gut microbiome to the phenotypic variation of muscle fatty acid contents

**Figure 6:**
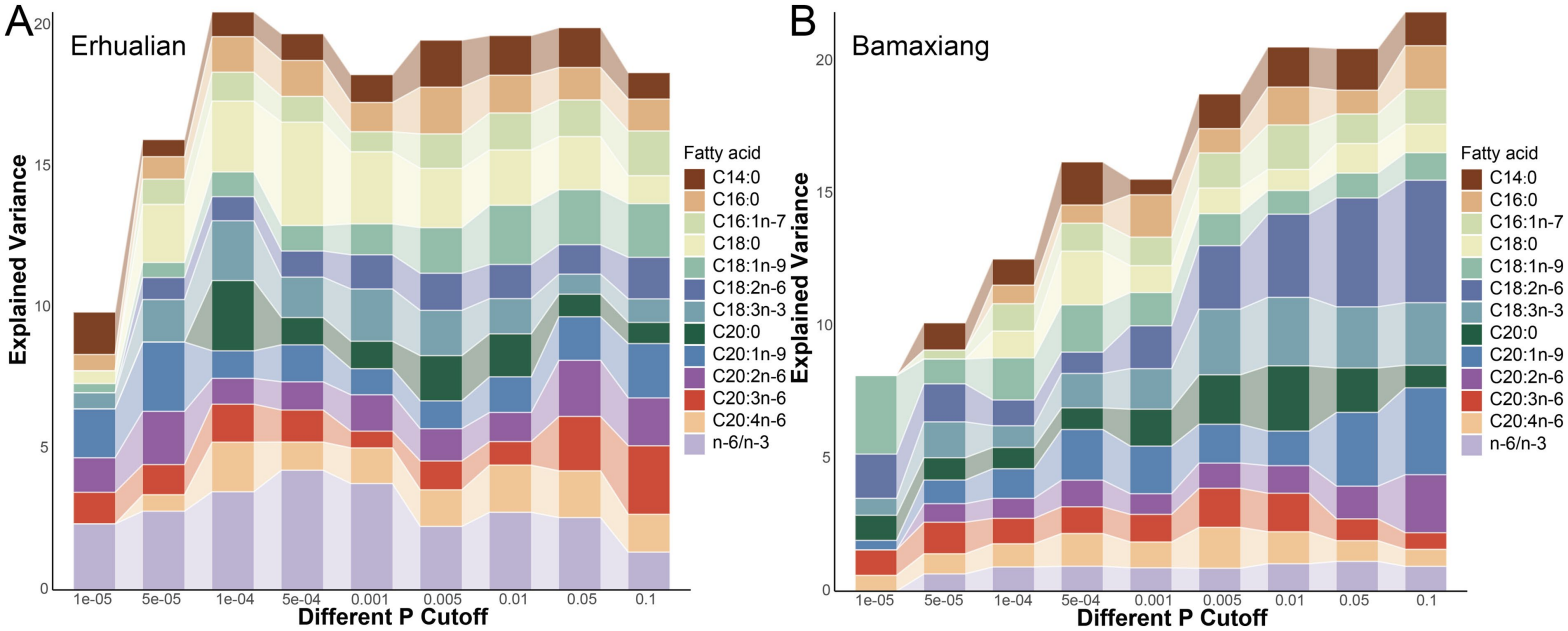
The phenotypic variance of muscle fatty acid contents explained by the gut microbiome at different significance levels in Erhualian **(A)** and Bamaxiang **(B)** pigs. The *x*-axis shows the different significance levels in identifying fatty acid-associated bacterial taxa, and the *y*-axis indicates the percentage of the phenotypic variance of muscle fatty acid contents.

To investigate the contribution of gut microbiome to the phenotypic variation of fatty acid contents. The 100 × cross-validation analysis was performed at different *p* value thresholds (1.0 × 10^−5^ to 0.1). At *p* = 1.0 × 10^−5^ level in Erhualian pigs, ASVs explained 0.45% phenotypic variation of stearic acid (C18:0) content, 0.32% of oleic acid (C18:1n-9), 0.59% of homolonolenic acid (C20:3n-6), and 1.34% of n-6/n-3 ratio. When the significance threshold of association was increased, more ASVs were included in the risk model, the explained variance increased to 3.66% for stearic acid (C18:0), 2.10% for oleic acid (C18:1n-9), 2.43% for homolonolenic acid (C20:3n-6), and 4.24% of n-6/n-3 ratio. For Bamaxiang pigs, the explained variance of muscle fatty acid contents by gut microbiome increased from 0.81 to 4.62% for linoleic acid (C18:2n-6), from 0.89 to 2.97% for oleic acid (C18:1n-9), from 0.36 to 3.28% for C20:1n-9, and from 0.0005 to 1.13% for n-6/n-3 ratio (Figure 6).

## 4 Discussion

Substantial researches in germ-free mice and humans have suggested that the gut microbiota not only affects host energy metabolism, but also has significant effects on fatty acid biosynthesis (Velagapudi et al., 2010; Rosenbaum et al., 2015). Short-chain fatty acids (SCFA) are linked with obesity and pathologies (Patloka et al., 2024). Gpr41, a G protein-coupled receptor expressed in the gut epithelium is a key regulator of the metabolism balance of host short-chain fatty acids depending on the gut microbiota (Samuel et al., 2008). However, whether the gut microbiota is related to the contents of muscle LCFAs has largely unknown. In this study, we performed the association analyses of gut microbiota with the contents of fatty acids in LD muscle with luminal content samples from Erhualian pigs and fecal samples from Bamaxiang pigs. We identified tens of significant associations and uncovered the significant contribution of gut microbiota to the variances of muscle fatty acid contents. The study provided important references for the production of pork with reasonable composition of fatty acids through regulating gut microbiota.

All experimental pigs from Erhualian and Bamaxiang cohorts were housed in the same farmhouse and fed identical formula diets. Samples were collected at 300 ± 3 days of age. Although Firmicutes and Bacteroidetes predominated in both breeds, Erhualian pigs exhibited reduced Firmicutes abundance relative to Bamaxiang pigs (*p* < 0.05), concomitant with elevated Bacteroidetes levels (*p* < 0.01). Distinct microbial compositional profiles emerged at the genus level, with these differences likely attributable to two key factors: sampling site (intestinal lumen vs. feces) and host genetic divergence between breeds, as established in prior studies (Benson et al., 2010; Kovacs et al., 2011).

LCFAs with carbon atoms from 14 to 20 are *de novo* synthesized in liver. Acetyl-CoA is a direct precursor of LCFA biosynthesis and mainly derived from fatty acid *β*-oxidation, pyruvate oxidative decarboxylation, and ketogenic amino acid metabolites in glucose metabolism. The whole-process of LCFA biosynthesis mainly includes the following three steps: (1) Acetyl-CoA is transported from the mitochondria to the cytoplasm; (2) Acetyl-CoA carboxylase catalyzes acetyl-CoA and CO_2_ to form malonyl-CoA; and (3) fatty acid synthesis cycle to synthesize palmitic acid (C16:0). Significantly, negative or positive correlations were found between LCFAs. This should reflect the interconversion between fat acids through desaturation/elongation reactions. Previous studies have also demonstrated that different fatty acids are not independent between each other. LCFAs can be transformed through desaturation or elongation processes (He Q. et al., 2023). Palmitic acid has been demonstrated to impact insulin secretion and regulate body weight (Benoit et al., 2009). In Erhualian pigs, we identified that *Lactobacillus*, *Lachnospira*, and *Fibrobacter* were associated with palmitic acid (C16:0). Furthermore, Erysipelotrichaceae and *Paludicola* were associated with palmitic acid (C16:0) in Bamaxiang pigs. Previous reports suggested that high abundance of *lactobacilli* and Erysipelotrichaceae was associated with host lipid absorptions (Yang H. et al., 2016; Zhong et al., 2022). It has also been found that *Fibrobacter* is one of the most important cellulose degrading bacteria in the gut. It could digest cellulose, cellobiose, and glucose, and produce succinate and acetate that are crucial for host nutrition and health (Froidurot et al., 2024).

In previous studies, n-6 PUFAs might promote inflammation by increasing the circulating linoleic acid (C18:2n-6) (Fritsche, 2008). Interestingly, a recent study on swine and mice models demonstrated that the influence of the gut microbiota on host fatty acid composition could be manipulated by oral administration of specific linoleic acid-producing microbiota (Wall et al., 2009). In Erhualian pigs, we identified that Desulfobacterota and *Campylobacter* were related to the content of 18 carbon fatty acids including linoleic acid (C18:2n-6) and linolenic acid (C18:3n-3). At the same time, Firmicutes, Proteobacteria, and Bacteroidota showed significant associations with n-6 PUFAs including linoleic acid (C18:2n-6), eicosadienoic acid (C20:2n-6), homolonolenic acid (C20:3n-6), and arachidonic acid (C20:4n-6). Our study also showed that *Oscillibacter* was associated with the content of arachidic acid (C20:0) in Bamaxiang pigs. *Oscillibacter* is capable of metabolizing cholesterol in the intestine, thereby helping to reduce cholesterol and cardiovascular disease risk (Li et al., 2024).

N-3 and n-6 PUFAs are defined by the position of the double bonds from the methyl end of the carbon chain. PUFAs can act as intermediates in signal transduction (Eyster, 2007), ingredients of eicosanoids (Fitzpatrick and Soberman, 2001) and proinflammatory factors (De Caterina et al., 1994). These polyunsaturated fatty acids should regulate immune responses and influence human cardiovascular (Yang L. G. et al., 2016). In this study, most of the bacteria related to n-6/n-3 ratio in luminant content samples of Erhualian pigs belonged to Lachnospiraceae, *Lactobacillus*, and *Ruminococcus*, which are abundant in the digestive tracts of many mammals and can hydrolyze starch and other sugars to produce butyrate and other SCFAs (Devillard et al., 2007; Biddle et al., 2013). Paludibacteraceae was found to be associated with n-6/n-3 ratio. Paludibacteraceae is a lignocellulosic decomposing bacterium that can utilize various soluble carbohydrates and monosaccharides, and produce acetate and propionate salts (Qiu et al., 2014).

Additionally, among eight n-6/n-3 ratio-associated ASVs obtained by both two-part model and co-abundance network analyses, one ASV was annotated to Muribaculaceae which is a type of highly active mucopolysaccharide degrading bacteria on the colon wall and can break down complex polysaccharides in food to produce SCFAs. The bacteria in Muribaculaceae play important roles in gut metabolic activity and are closely related to host weight regulation and obesity (Lee et al., 2019; Pang et al., 2022). Another ASV was annotated to Lachnospiraceae. Many species in Lachnospiraceae have been reported to be involved in bile acid conversion and SCFA synthesis (Lin et al., 2024). One n-6/n-3 ratio-associated ASV belonged to Oscillospiraceae. Oscillospiraceae has been reported to be associated with a decreased incidence of obesity and obesity-related diseases, and showed the ability to ferment complex carbohydrates (Zhao et al., 2022; He C. et al., 2023).

Evaluating the association of gut microbiota in the same gut location of both pig populations with LCFA contents in pork are the optimal experimental design for this study. However, it was sometimes difficult to take into account the sampling from multiple gut locations of experimental pigs in the large-scale slaughter measurement. These two pig populations were raised and slaughtered in different years. We could not harvest cecum lumen samples from Bamaxiang pigs because of the slaughter procedure. However, it provided us another opportunity to evaluate differential effects of microbial compositions between feces and cecum on muscle LCFA contents. Distinct LCFA-associated bacterial taxa between Erhualian and Bamaxiang pigs should be due to that (1) microbial samples were collected from different gut locations in two pig populations. Different numbers of specific LCFA type-associated bacterial taxa were detected between cecum luminal content and fecal samples (Figure 3A), suggesting that intestinal location heterogeneity existed for the specific LCFA type-associated bacterial taxa. For example, significantly more n-6/n-3 PUFA ratio-associated ASVs were identified in cecum luminal content samples than in fecal samples. This indicated that intestinal location heterogeneity should be considered when we established the regulation methods for gut microbiota to improve the content of specific LCFA type; (2) different pig breeds were used. Host genetics should play important roles in the gut microbial compositions; (3) There might be a little difference in the nutrient requirement of these two Chinese indigenous pig breeds, which affected both muscle LCFA contents and gut microbial compositions. However, the commercial formula diet provided to both experimental pig populations were produced according to the standard of “The Nutrient Requirements of Swine” (GB/T 39235–2020). It specifies the nutrient requirements for all lean-type pigs, fat-type pigs, and meat-fat-type pigs. Moreover, we observed the similar fatty acid contents in LD muscle in two pig breeds.

The functional prediction analysis with PICRUSt2 demonstrated that the KEGG pathways related to the metabolisms of carbohydrate and lipid, and the pathway of fat digestion and absorption were positively associated with the contents of muscle fatty acids in luminal content samples of Erhualian pigs, suggesting that metabolites of carbohydrate and lipids might provide precursors for the biosynthesis of LCFAs. However, adipocytokine signaling pathway and thermogenesis were negatively associated with muscle fatty acid contents. As we have well known, LCFAs can be used to synthesize lipids, and thermogenesis requires the consumption of fatty acids (He Q. et al., 2023). Similar to the LCFA-associated CAGs, only few LCFA-associated functional pathways were identified, indicating the advantage using cecum content samples for testing the association between gut microbiome and muscle fatty acid contents. However, as a limitation, functional prediction based on 16S rRNA gene sequencing data only provided a reference for potential functional capacities of gut microbiome. Further metagenomic sequencing and metatranscriptome analysis should be performed for further accurately elucidating the functions of gut microbiome involved in the muscle LCFA contents.

We evaluated the contribution of the gut microbiome to the variation of muscle contents of twelve fatty acids and found that it explained 1.47–4.62% of the phenotypic variation. Compared to previous reports, we found that the gut microbiome showed a less contribution to the fatty acid contents in pig muscle than pig genetics (quantitative trait loci) (Yang et al., 2013). This may be caused by the reason that all experimental pigs were raised in the same farmhouse and provided with the same diet, which reduced the impact of environmental factors including gut microbiome on muscle fatty acid contents. However, the results from this study suggested the potential effect of gut microbiome on fatty acid contents in pig muscle.

In summary, we observed the significant association between the gut microbiota and fatty acid contents in longissimus dorsi, and identified tens of LCFA-associated bacterial taxa and potential functional pathways. We found that gut microbiome could explain 1.47–4.62% of the phenotypic variation of muscle fatty acid contents, and suggested that intestinal location heterogeneity existed for the specific LCFA type-associated bacterial taxa. The limitations of this study mainly included: (1) only 16S rRNA gene sequencing was performed. It provided low resolution to the gut microbial composition. (2) microbial samples were collected from different gut locations in two pig populations. This made it difficult to compare the results of LCFA-associated bacterial taxa between two pig populations. However, we observed the similar fatty acid contents in LD muscle of two pig breeds and all pigs were raised in the same farm house. The results from this study provided important insights about the contribution of gut microbiome to the variation of muscle fatty acid contents, and gave the basic knowledge for improving the muscle fatty acid contents by regulating the gut microbiome.

## Data Availability

The 16S rRNA gene sequencing data generated in this study were deposited in NCBI Sequence Read Archive (SRA) under accession numbers: SRR4422912, SRR4422947, SRR4422914, SRR4422951, SRR4431318, SRR4431319, SRR4431321, SRR4454082, SRR4454119, and SRR4431322.

## Supplementary material

SUPPLEMENTARY FIGURE 1 The correlation between the β-diversity of gut microbiota and long chain fatty acid contents in longissimus dorsi muscle. **(A)** Erhualian pigs. **(B)** Bamaxiang pigs.
