# Supplementary figures and images for: Uncovering the heterogeneity of the gut microbial taxa associated with the contents of different fatty acids in muscle with cecum luminal content and fecal samples from two pig populations
Source: Front Microbiol. 2025 Apr 30;16:1575383. doi: 10.3389/fmicb.2025.1575383 (PMC12075296; doi:10.3389/fmicb.2025.1575383)

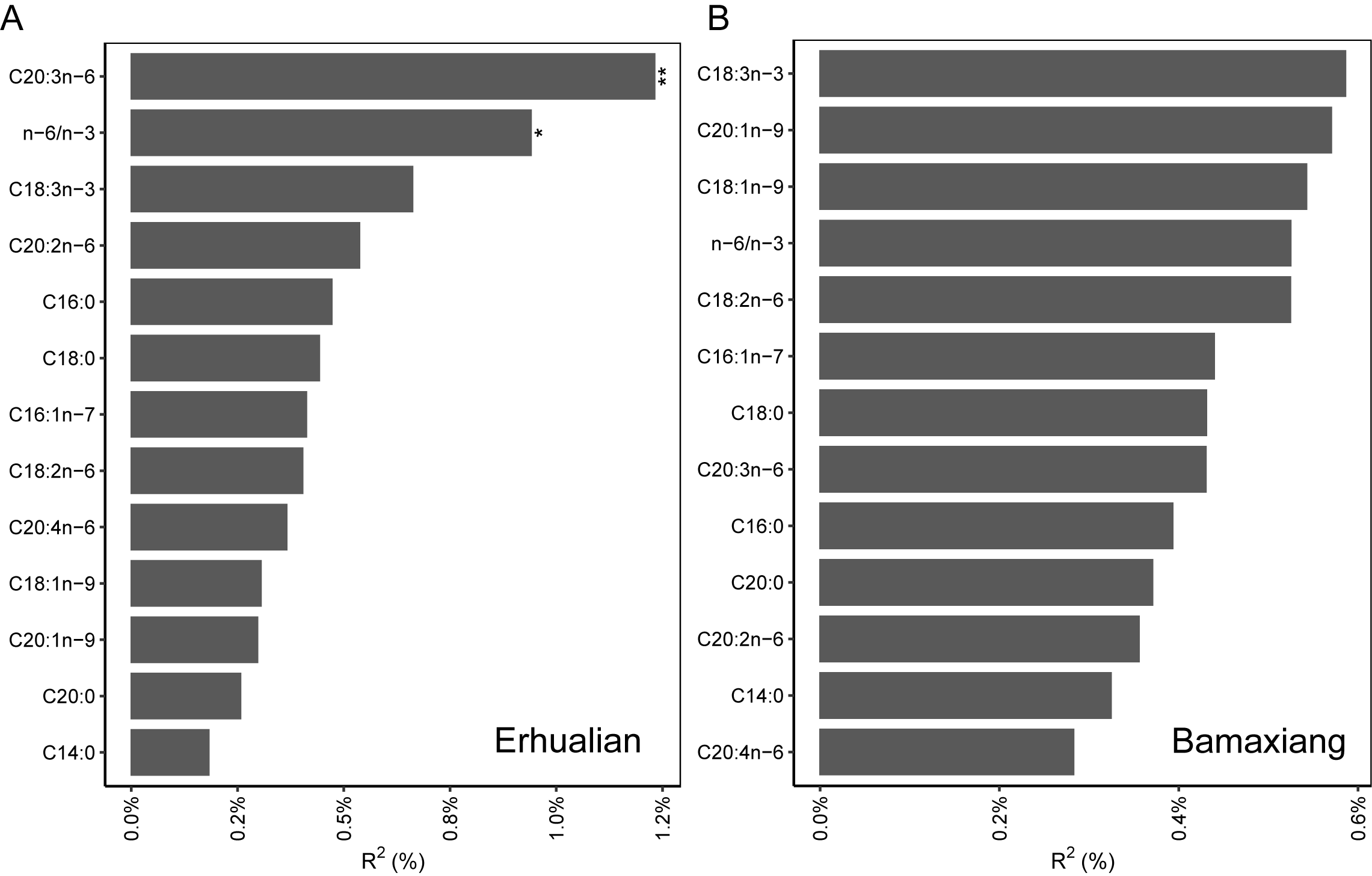

Supplement: SUPPLEMENTARY FIGURE 1 — The correlation between the β-diversity of gut microbiota and long chain fatty acid contents in longissimus dorsi muscle. (A) Erhualian pigs. (B) Bamaxiang pigs. [file Image_1.tif]
